# Effect of ultraviolet light-emitting diode processing on fruit and vegetable-based liquid foods: A review

**DOI:** 10.3389/fnut.2022.1020886

**Published:** 2022-11-29

**Authors:** Fernando Salazar, Sebastián Pizarro-Oteíza, Ismael Kasahara, Mariela Labbé

**Affiliations:** Escuela de Alimentos, Pontificia Universidad Católica de Valparaíso, Valparaíso, Chile

**Keywords:** UV-LED (ultraviolet light-emitting-diode array), liquid food, enzymes, microorganisms, nutritional and functional properties, computational simulation

## Abstract

Ultraviolet light-emitting diode (UV-LED) technology has emerged as a non-thermal and non-chemical treatment for preserving liquid fruit and vegetable foods. This technology uses ultraviolet light to interact with the food at different wavelengths, solving problems related to product stability, quality, and safety during storage. UV-LED treatment has been shown to affect microbe and enzyme inactivation, and it increases and improves retention of bioactive compounds. Moreover, computational simulations are a powerful and relevant tool that can be used optimize and improve the UV-LED process. Currently, there are a limited studies of this technology in liquid fruit and vegetable-based foods. This review gathers information on these food type and shows that it is a promising technology for the development of new products, is environmentally friendly, and does not require the addition of chemicals nor heat. This is relevant from an industrial perspective because maintaining the nutritional and organoleptic properties ensures better quality. However, due to the scarce information available on this type of food, further studies are needed.

## Introduction

Vegetables and fruits are rich in phytonutrients, vitamins, minerals, carotenoids, antioxidants, polyphenols, and dietary fibers, all of which are essential to the health of the human body (1–3). Recent research has shown that regular vegetable intake is associated with a reduction in cardiovascular disease and cancer (4, 5). The consumption of fruits and vegetables has increased in correlation with a growing consumer awareness about a nutritious and balanced diet (6). Although the total production and processing of fruits and vegetables has multiplied, there has also been an increase in food loss in the form of waste and by-products. The Food and Agriculture Organization of the United Nations (FAO) has estimated loss and waste of fruits and vegetables at up to 60% of the total horticultural production (6, 7). Nowadays, the conventional form of processing and preservation for many fruit and vegetable juices is heat treatment. However, this process causes negative effects on the organoleptic, physical and chemical properties, as well as the nutritional quality (8). The increasing availability of minimally processed fresh produce sets a high quality standard; these products are fresher and preserve the vitamin, mineral, fiber and antioxidant content for a more nutritional product (9). The loss of nutrients which occurs in conventional processing and the growing demand of consumers for high quality food products have motivated the search for alternative processing methods, especially modern and non-thermal technologies (10, 11). The use of ultraviolet light (UV) for processing liquid fruit and vegetable foods continues to increase in popularity due to its non-thermal and non-chemically invasive nature (12). In addition, it has been widely used as a tool for quality control in agricultural fields (13). Ultraviolet light is defined as any light in the electromagnetic spectrum with a wavelength between 100 and 400 nm (12). UV technology is being increasingly applied in food processing and is highly effective against most pathogens, easy to operate and does not require the addition of chemicals (14).

In industrial processing, UV technology has been successfully implemented in disinfection of water, air and smooth surfaces, and is used for various liquid and solid foods, such as fresh produce, and fruit juices, among others (12). Likewise, Song et al. (14) stated that UV application has grown rapidly and that the most widely used ultraviolet light source for disinfection is mercury based lamps. Despite having high optical power and germicidal efficacy, mercury lamps also have several disadvantages including: possible food contamination with mercury, brittle materials, and they require time to warm up before they reach their maximum optical power (12).

Currently, ultraviolet light-emitting diode (UV-LED) irradiation is being considered as a new source of ultraviolet light and has gained interest as an alternative to conventional UV (14, 15). In addition, UV-LED lamps are advantageous because of their compact size, low power consumption and long shelf life (50,000–100,000 h) compared to conventional UV lamps (15,000 h) (16, 17).

Studies show an increase in antioxidant properties, enzyme and microbiological inactivation where lamps have been implemented in fruit and vegetable juice production (5, 12, 17–20). Furthermore, researchers are beginning to use mathematical modeling combined with computational simulation of UV-lamps’ effect on produce. Mathematical modeling allows for prediction of transport phenomena in food processing, which will inform better equipment design and improve process control methodology (21).

Lemus-Mondaca et al. (22) expresses that process prediction is a tool to improve processes and minimize problems such as excessive energy consumption and product damage, among others. To obtain the optimal effects from UV-LED irradiation processing, it is necessary to investigate the interaction of the complex phenomena involved including hydrodynamics, irradiation, and kinetics (23). Nevertheless, more studies are needed to fully understand the effect and application of UV-LED irradiation for fruit and vegetable-based liquid foods. Therefore, the main objective of this review was to collect information on the effects and applications of UV-LED irradiation, such as microbiological and enzymatic inactivation, retention of functional compounds, and computational simulation of fruit and vegetable processing in order to provide relevant knowledge about this technology.

## Ultraviolet light technology in liquid food

### General

Initially, UV radiation technology was used for disinfecting drinking water and sterilization of medical devices. In water treatment, UV radiation is efficient in the inactivation of chlorine-resistant protozoa. The successful implementation of this process provides a tool for processing various food products such as fruit juices, and vegetables, among others (24). Current research on this technology in the food industry is in development and continues to grow in popularity due to its non-thermal and chemically non-invasive nature (12). Ultraviolet light has been used since the beginning of 20th century, while according to Kheyrandish et al. (25), UV light has been used for the last two decades for wider treatment purposes, such as disinfecting fruit and vegetable-based liquid foods.

### Classification

Traditional sources of ultraviolet irradiation (UV) are low and medium pressure mercury lamps (14). To properly apply the technology, lamps must be classified according to their operational wavelengths, as stated by Koutchma, (24) and Hinds et al. (26).

Ultraviolet light is classified into four categories: long-wave UV-A, with wavelengths of 315–400 nm; medium wave UV-B, with wavelengths 280–315 nm; short-wave UV-C, with wavelengths between 200 and 280 nm; and vacuum wave UV-V with wavelengths of 100–200 nm (24). Among these types of UV light, UV-C light is the most widely applied, especially in water, air, and surface disinfection (27, 28). Furthermore, according to Guerrero-Beltran and Ochoa-Velasco (28), UV-C technology has been studied considerably in fruit and vegetable-based liquid foods and several studies have been carried out with the aim of inactivating both native flora and inoculated microorganisms, and to analyse the behavior of enzymes and bioactive compounds (vitamin C, polyphenols, and antioxidants). Table 1 summarizes this information.

**TABLE 1:** Summary of studies related to ultraviolet (UV-C) processing of liquid fruit and vegetable-based foods.

Liquid food	UV dose	Reactor type	Results	References
				
Grape juice	78.56 mJ/cm^2^	Dynamic	Log reduction of 1.6 for lactic acid bacteria	(70)
Pineapple juice	21.52 mJ/cm^2^	Dynamic	Log reduction of 1.4 for yeast plus molds	(71)
Apple cider	14 mJ/cm^2^	Dynamic	Log reduction of 2.0 for *E. coli* O157:H7	(72)
Carrot-orange juice	1,060 mJ/cm^2^	Dynamic	Log reduction of 2.6 for *S. cerevisiae* KE162	(73)
Orange juice	144 mJ/cm^2^	Dynamic	82% retention in vitamin C	(74)
Mango juice	0.35 mJ/cm^2^	Static	8% retention in vitamin C, increase 31% of total phenolic compounds and total flavonoids and 12% of antioxidant capacity	(61)
Starfruit juice	0.22 mJ/cm^2^	Dynamic	Increase 3.3% of antioxidant capacity	(75)
Black carrot juice	21.6 mJ/cm^2^	Static	Decrease 35.3% of PME residual level	(76)
Pitaya juice	102.6 mJ/cm^2^	Dynamic	Decrease 11.6% of total phenolic compounds	(77)
Apple juice	21.9 W/m^2^	Static	Polyphenol oxidase enzyme decrease 80%	(78)
Tropical juice	689 J/L	Dynamic	Reduced to 3.1 log for yeast plus molds	(79)
Watermelon juice	9,685 J/L	Dynamic	Pectin methylesterase activity decrease 65% compared to heat treatment	(80)
Orange juice	918 J/L	Dynamic	Reduced to 1.85 log for aerobic plate count (APC)	(79)
Watermelon juice	37.5 J/L	Dynamic	Reduced to 1.5 log for total plate counts	(81)
Apple and grape juices	100,480 J/L	Dynamic	PFO residual activity in apple juice decreases to 15.8% and in grape juice to 61%	(82)

Effect on microorganisms, bioactive compounds (vitamin C, polyphenols, flavonoids, and antioxidants), and enzymes.

### Extrinsic and intrinsic factors

The factors that affect the efficacy of UV liquid food processing according to Hinds et al. (29) are both intrinsic, such as the food absorption coefficient, and extrinsic, such as the design of the UV reactor.

The intrinsic factors that affect UV liquid food processing relate directly to the way light is absorbed by different liquid foods and this behavior will vary according to the absorption coefficient (29). According to Choudhary and Bandla (30) the absorption coefficient is determined by the food’s optical properties. Guerrero-Beltrán and Barbosa-Cánovas (31) stated that the propagation capacity of UV light decreases as the absorption coefficient increases and that this parameter is directly proportional to color and turbidity of the liquid. In order to obtain efficient UV treatment of liquid foods, it is necessary to take into account the presence of suspended particles and soluble organic molecules that absorb and scatter UV light (32–34).

**FIGURE 1:**
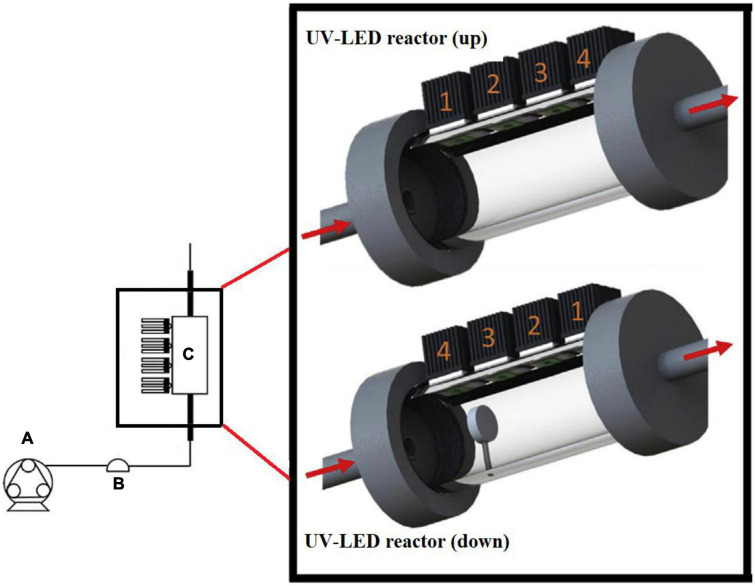
Scheme of the flow-through ultraviolet light emitting diode (UV-LED) reactor setup: **(A)** Peristaltic pump, **(B)** Pulse dampener, **(C)** UV-LED reactor (up/down). Adapted from Keshavarzfathy et al. (23).

Regarding extrinsic parameters such as reactor configuration and design (Figure 1), Hinds et al. (29) mentions that the following must be determined: type of light exposure (continuous or pulsed), UV light source, conditions during treatment (temperature/light or darkness), distance of the sample from the light source, reactor type (dynamic/static), design of the irradiation system including flow rate, number of lamps, irradiation exposure time, dose or fluence, irradiance, and fluence rate.

### Common concepts

In order to study an efficient processing of ultra violet light in fruit and vegetable-based liquid foods, it is necessary to understand the concepts at play when light interacts with these foods (29, 35, 36). The authors explain this technology with vocabulary that ensure the correct application of UV irradiation. These concepts are commonly used in the field of photochemistry (37). The most relevant concepts are Source Radiant Power, Source Radiant Energy, Dose or Fluence, Irradiance, and Fluence Rate.

• Source Radiant Power is the total radiant power emitted in all directions by a radiant energy source (units, W).
• Source Radiant Energy is the total radiant energy emitted from a source over a given period of time (units, J).
• Irradiance (E) refers to the radiant flux or radiant power (P) of all wavelengths incident on an infinitesimal element of surface containing the point under consideration divided by the area (S) of the element. Furthermore, for a parallel and perpendicularly incident beam not scattered or reflected by the target or its surroundings, fluence rate (E0) is an equivalent term (units, mW/cm^2^).
• Fluence Rate (E0) is quadruple the ratio of the radiant power (P) incident on a small spherical volume element and containing the point under consideration, divided by the surface area of that sphere. Units are mW/cm^2^.
• Dose or Fluence (H0) refers to the total radiant energy traversing a small transparent imaginary spherical target containing the point under consideration, divided by the cross section of this target. Additionally, dose equals the Fluence Rate multiplied by the irradiation time in seconds (units, mJ/cm^2^).

## Ultraviolet light-emitting diode processing in fruit and vegetable-based liquid foods

### General

The availability of fruit and vegetable-based liquid foods has increased due to the growing interest in health and wellness in the last 20 years (19, 38). Fruit and vegetable-based liquid food are rich sources of minerals, vitamins, and various functional bioactive compounds that contribute to a healthy diet (39). However, these foods are highly susceptible to microbial spoilage, so processing is necessary to preserve them and improve their food quality.

Conventional techniques to preserve fruit and vegetable-based liquid foods are thermal and chemical treatments, however, their use can lead to the loss of desired organoleptic properties and thermolabile compounds (26). In addition, conventional termal processes such as pasteurization, sterilization, evaporation, refrigeration, freezing, and drying are the highest energy consuming technologies in the food industry (40). As such, new non-thermal processing technologies are being developed in order to ensure safe and high-quality food, with long shelf life and minimized environmental impact (26).

The application of UV-C light for non-thermal pasteurization of liquid foods has been studied extensively (29). However, Popović et al. (12) stated that there are few studies using UV-LEDs for fruit and vegetable-based liquid foods. As such, the information available on the effects of UV-LED irradiation processing on the quality, functional, and nutritional properties of liquid foods is limited. Meanwhile, there are related studies on microbe and enzyme inactivation, and on the functional properties of fruit and vegetable-based liquid foods (5, 18, 19, 41, 42).

The properties of LED light generated special interest for further investigation at the end of the 1960s, when LEDs were in the early stages of development. At this time they were low-powered and mainly used as indicator lamps (17). UV-LED allows improved manipulation of light-time settings, radiant intensity and spectral characteristics compared to UV light from low-pressure mercury lamps (43). Subsequently, the development of LEDs was rapid as new semiconductor materials were incorporated, crystal growth techniques were improved, and better means of thermal dissipation were implemented (43, 44). LED technology is a semiconductor that emits light when electricity passes through it and works according to the principle of electroluminescence, that is, LEDs produce light by applying an electric or magnetic field. The excited electrons reach a state of lower energy when emitting light, and release energy in the form of electromagnetic radiation (45).

## Applications

### Microbiology inactivation

The effect of ultraviolet light in fruit and vegetable-based liquid foods processing on microbial population and product quality has been studied (46). According to Popović et al. (12) the most important advantage of LEDs is the ability to combine multiple wavelengths of light to improve microbial treatment efficacy and trigger a synergistic inactivation of microorganisms. To generate this effect is necessary to understand what Prasad et al. (45) has explained. These authors mention that LED light acts on the bacterial cell wall by exciting light-sensitive compounds, one of which is porphyrins. These excited compounds collide and transfer energy to oxygen molecules, producing reactive oxygen species (ROS) such as hydroxyl radicals and hydrogen peroxide, which react with cellular components and cause cell death (47). The inactivation mechanism caused by UV-C irradiation is based on the absorption of photons by the genetic material and the formation of pyrimidine dimers that inhibit cell transcription and replication (24, 48). Furthermore, pyrimidine dimers that inhibit cell transcription and replication of different microbial groups such as enteral bacteria, cocci and micrococci, spore formers, enteric viruses, yeasts and fungi have been studied under the effect of UV-C (24, 48, 49). UV-C energy reacts photochemically with DNA and wavelengths between 250 and 260 nm have been shown to be efficient in absorbing UV light in DNA (50). However, Popović et al. (12) stated that the most efficient wavelength is 280 nm.

Therefore, wavelength plays an important role in microbial inactivation (Table 2). On the other hand, studies on microbial inactivation by UV-LED irradiation in fruit and vegetable-based liquid foods are only recently emerging, of which; (51) reported reduction values of 0.4 and 1.6 log for *E. coli* DH5 α in commercial orange juices treated with UV-A-LED at 365 nm with 147 J/cm^2^.Akgün and Ünlütürk (18) showed results in apple juice for a static reactor equipped with four LEDs (254, 280, 365, and 405 nm), but did not provide a log reduction of *E. coli* K12. However, they found that the combined effect of 280/365 nm wavelength was more influential in the inactivation of *E. coli* K12 than single wavelengths. Baykuş et al. (19) tested UV-LED using emissions at 280, 365, and 280/365 nm for the inactivation of *E. coli* K12 in a mixed beverage of carrot, carob, ginger, grape, and lemon juice, and concluded that treatment at 280 and 280/365 nm resulted in greater log reductions of *E. coli* K12 than those treated at a wavelength of 365 nm. Lian et al. (41) also analysed the behavior of UV-LED in orange juice and concluded that when using a 365 nm wavelength and fluence rate of 126 J/cm^2^, the *E. coli* DH5α bacterium was reduced from 0.35 to 1.58 on a logarithmic scale.

**TABLE 2:** Mechanism of microbial inactivation for ultraviolet (UV-A, UV-B, and UV-C) ranges (12).

UV	Wavelength (nm)	Mechanism in microbial inactivation
		
A	320–400	Causes increased production of reactive oxygen species (ROS), resulting in oxidative damage to membrane (lipids and proteins) and indirect effects on DNA.
B	280–320	Induces direct DNA damage by forming photo products that obstruct DNA replication and RNA transcription, oxidative stress, and lipid damage.
C	200–280	Causes direct DNA damage by inducing the formation of pyrimidine dimers that obstruct DNA replication and RNA transcription. On the basis of the above, the most efficient region is for 280 nm.

### Enzymatic inactivation

Ultraviolet light radiation is considered to be a new and environmentally friendly technology for extending the shelf life of fruits and vegetables. However, most studies of this technology in vegetables and fruits have focused on microbe inactivation (52). Therefore, it is necessary to investigate other UV sources, such as LEDs and different characteristics such as enzyme activity, which is also relevant in vegetable processing.

Fruit and vegetable-based liquid foods such as juices are susceptible to enzyme activity that limit their shelf life. Enzymes such as pectinmethylesterase (PME), polygalacturonase (PG), polyphenol oxidase (PPO) and peroxidase (POD) are found in such products and affect stability aspects as they react uncontrollably (28). For example, enzymatic browning is caused by oxidative enzymes (52) and the cloudy appearance of some fruit juices can be lost due to phase separation caused by pectolytic enzymes (53).

Ultraviolet irradiation has been studied for the inactivation of tyrosinase and causes of oxidation of SH groups, which leads to changes in native protein conformations by forming cross-links between polypeptides (54). Since enzyme activity depends on their structure, oxidation of SH groups leads to their inactivation. Furthermore, authors such as Falguera et al. (52) and Jiang et al. (55) stated that the management of the tyrosinase enzyme is a critical challenge in the handling and storage of horticultural products. Fruit juices naturally contain some compounds that protect these enzymes from being denatured by the irradiation process (24, 31).

Control of these enzymes is important for delivering products with high organoleptic, functional, and nutritional quality. Another argument used is the limited number of studies on the enzymatic activity of fruit and vegetable-based liquid foods processed with UV-LED. Here, we can highlight Akgün and Ünlütürk (18) who showed effective inactivation of PPO in apple juice using LEDs at UV-C/UV-A with a residual activity of 32.6%, and after using a combination of UV-C/405 nm with a dose of 771 mJ/cm^2^ residual PPO activity was 34.4%. Meanwhile, Pizarro-Oteíza and Salazar (20) mentioned that the UV-LED processing efficiently inactivated pectinases [Pectin methyl esterase (PME) and polygalacturonase (PG)] in tomato juice and could be used as an alternative to thermal treatments (cold break and hot break). Tomato juice treated with UV-LED (117 mJ/cm^2^) obtained a similar PME activity to that of samples treated by cold break, and with 351 mJ/cm^2^ obtained a PME activity 28.3% lower than samples treated by hot break. In addition, UV-LED generated a (PG) activity similar to that of hot break and 49% lower than that of cold break.

However, the inactivation of enzymes that degrade the quality of fruit and vegetable-based liquid foods depends on both the intensity of the irradiation, and in particular the exposure of the emitted UV light, among others (18, 52).

### Functional compounds

Ultraviolet light emitting diodes in the food industry are used in three main areas: food production, post-harvest storage, and food safety (17, 18). Furthermore, this technology is one of the most promising alternatives for extending the shelf life of fruits and vegetables (56). Studies have been published showing the efficiency of LEDs in post-harvest applications, where LEDs were able to accelerate secondary metabolites during the ripening process (57), and both increase the volume and delay the loss of nutritional content in fruits and vegetables such as broccoli, citrus fruits, and strawberries (57–59). However, UV-LED studies on liquid fruit and vegetable foods are only starting to emerge.

Popović et al. (12) concluded that UV-LED treatment of a mixed fruit drink increased total phenolic compounds 1.7-fold, antioxidant capacity 4.6-fold and preserved total carotenoid content better than conventional heat treatment. Bhat (60) and Santhirasegaram et al. (61) explained that the increase in total phenols can be attributed to depolymerization of polyphenol polymers, release of conjugated phenolic compounds bound to cell wall polysaccharides, and inactivation of polyphenol oxidase. In contrast, Xiang et al. (5) found that UV-C-LED generated negative impacts on some quality characteristics of apple juice, such as a decrease in total phenolic content, antioxidant activity, and change in color. However, this technology could still be considered a valid alternative to conventional preservation methods in fruit and vegetable-based liquid foods if certain UV-C-LED processing conditions were optimized or combined with other methods. On the other hand, Akwu et al. (62) showed that polyphenols and vitamins with UV-C-LED irradiation in apple juice induced significant reductions at the conditions evaluated. Riboflavin was relatively stable. Epicatechin and chlorogenic were significantly reduced at high exposure doses. In contrast, minor changes were observed at the 40 mJ/cm^2^ dose. With respect to ascorbic acid it was reduced up to 32%, at the 160 mJ/cm^2^ dose while a 17% reduction was observed at 40 mJ/cm^2^.

These results support the idea of deeper studies, not only from a food safety perspective, but also to better characterize of the effect of the UV-LED irradiation process on the nutritional and functional quality of fruit and vegetable-based liquid foods.

### Computational simulation

Currently, many countries have seen an increase in the production of fruit and vegetable-based liquid foods. Research, development, and technological innovation in food processing have been used to achieve better food quality and deliver products with high nutritional and functional quality. In addition, the challenges of the environmentally friendly food industry require high productivity, as well as efficient and optimized processes (63). Mathematical modeling combined with computational simulation allows for the prediction of transport phenomena in food processing, which will lead to better equipment design and an improvement in process control (21).

The determination of the radiant energy profile is essential for the design and optimization of UV reactors. To simulate the radiation distribution, it is necessary to consider the geometry of the ultraviolet (UV) source and its characteristics, and the propagation of the rays through different media and the reflection of the relevant surfaces. In addition, Pan et al. (64) stated that to be able to form a reliable mathematical prediction it is necessary to define some concepts such as:

A) Geometric model: The physical model of UV disinfection reactor

B) Computational mesh: Simulation space

C) Mathematical model

C.1. Flow model: Laminar or turbulent

C.2. UV radiation model: The model solves the propagation equation for radiation emitted from a finite solid angle and each angle corresponds to a fixed direction of the coordinate system.

C.3. Radiation dose calculate method: UV dose is an important parameter to evaluate the effectiveness of UV reactor.

To obtain the optimal benefit of UV-LED processing it is necessary to investigate the interaction of complex phenomena that are involved such as hydrodynamics, irradiation and reactor performance kinetics (23). In addition, developing a model to predict the fluence and irradiance distribution on UV-LED regardless of its structure and radiation profile is one current study (65). The predicted fluence rate and irradiance were validated using experimental actinometry and chemical radiometry data, respectively, and the model was used to quantify some assumptions of the UV-LED radiation profile. In addition, this model was linked with computational fluid dynamics (CFD) software to predict the fluence rate distribution inside the UV-LED reactor. The main application of this model is the prediction of the creep rate on the plate surface where the liquid sample is located (66–68).

This software can also be used to efficiently model the irradiation and kinetics of the reactor, and to understand specific characteristics such as irradiation patterns and polychromatic irradiation as proposed by Keshavarzfathy and Taghipour (23) and Wols et al. (69). These authors stated that particle tracking, residence time and contact time in disinfection reactors were correctly modeled by CFD software. Since there is currently no physical method available to specifically measure this phenomenon, a validated optimization model is needed to predict the distribution of irradiation in nearby fields. Furthermore, residence time distributions, which characterize fluid hydraulics, are critical factors to produce a certain geometry of the UV-LED reactor.

Hence, with tools to accurately predict the behavior of the particles, one can improve the hydraulics and thus increase the UV-LED effect (69). The integrated reactor model is informed by experimental studies with empirical data on the UV-LED effect in food matrices under different operating conditions such as flow rates, flow regimes, radiant powers and UV-LED configurations. Agreement between numerical predictions and existing experimental data demonstrates the ability to simulate the performance of the UV-LED reactor for future experimental and industrial applications.

Finally, the accurate prediction of UV-LED processing is related to the correct design of the equipment or prototype according to the process flow conditions. Therefore, a good UV-LED reactor design combined with an optimal computational simulation will guarantee an efficient UV-LED application which could be applied to different fruit and vegetable-based liquid foods.

## Future perspectives

Food and drug administration (FDA) approval of UV light as an alternative treatment to thermal pasteurization of products such as fresh juice has contributed to increased interest in this technology (24). Based on the above, UV-LED technology is a new UV source that has gained prominence in recent years (5, 12, 17–19). This technology allows for better manipulation of light time settings, radiant intensity, spectral characteristics, and delivers better thermal dissipation compared to UV light from low-pressure mercury lamps (43, 44). However, as stated by Popović et al. (12) low optical power is the main obstacle to the application of LEDs (UV-C and UV-B) on a commercial scale. Until optical power catches up with mercury lamps, industrial food applications of LEDs will likely involve low long-term UV exposure.

Currently, numerous investigations are being carried out on the use of UV-C to inactivate microorganisms in liquid foods and have delivered results which range from an experimental to a larger scale approach. Based on the above, a correct design of the UV reactor can reduce the interference of absorption and UV power (12, 23, 24). Moreover, with the right equipment design, not only could the microbial load be reduced, but the effects on bioactive compounds, enzymatic activities, organoleptic properties, and other properties of liquid fruit and vegetable-based foods could be studied in a controlled manner. Finally, it is important to recognize what Koutchma, (24) brought up with respect to the applied preservation process, because in order to provide a thorough analysis, validation studies of the food and processing are needed. That is, more studies are required to clarify the detailed behavior of each phenomenon and/or application attributed to UV-LED technology on fruit and vegetable-based liquid foods.

## Conclusion

Non-thermal processing methods are fast, efficient, and reliable alternatives for improving the quality of fruit and vegetable liquid foods. We conclude that UV-LED irradiation is an attainable technology with the potential to become an economic and commonplace method for processing fruit and vegetable juices. Furthermore, this technology has positive effects on microbe and enzyme inactivation in these products and has a high potential for increasing and retaining bioactive components. On the other hand, it will be interesting to increase our knowledge of prediction models and computational simulation, in order to optimize food processing and correlate theoretical data with empirical values. Finally, more research is needed to give more clarity about the effects of this technology on fruit and vegetable-based liquid foods.

## Author contributions

FS: idea, funding, and review of the manuscript. SP-O: structure, drafting, and response to reviewers of the manuscript. IK and ML: review and response to reviewers of the manuscript.
